# Biomechanical parameters of the cornea measured with the Ocular Response Analyzer in normal eyes

**DOI:** 10.1186/1471-2415-14-11

**Published:** 2014-01-30

**Authors:** Aachal Kotecha, Richard A Russell, Angelos Sinapis, Sayeh Pourjavan, Dimitros Sinapis, David F Garway-Heath

**Affiliations:** 1 NIHR Biomedical Research Centre for Ophthalmology, Moorfields Eye Hospital NHS Foundation Trust and UCL Institute of Ophthalmology, 11-43, Bath Street, London EC1V 9EL, UK; 2 St Luc University Hospital, Université Catholique de Louvain, Brussels, Belgium

## Abstract

**Background:**

To evaluate the relationships between Reichert Ocular Response Analyzer (ORA) parameters corneal hysteresis (CH) and corneal response factor (CRF) and ocular dimensions, age and intraocular pressure.

**Methods:**

Two hundred and twelve eyes of 212 participants with no ocular pathology had CH and CRF measured with the ORA. Intraocular pressure (IOP) was measured with the Dynamic Contour tonometer and central corneal thickness (CCT) was also evaluated. Partial least squares linear regression (PLSLR) analyses were performed to examine the relationships between each response variable, CH and CRF, and the predictor variables age, corneal curvature (CC), axial length (AL), CCT and IOP.

**Results:**

CH was positively associated with CCT and negatively associated with age (scaled coefficients: CCT 0.62, p < 0.0001; age -0.55, p <0.0001; r^2^ = 0.25). CRF was positively associated with CCT and DCT IOP and negatively associated with age and AL (scaled coefficients: CCT 0.89, p < 0.0001; DCT IOP 0.46, p < 0.01; age – 0.60, p < 0.0001; AL -0.37, p < 0.01; r^2^ = 0.43). There was no significant association between CC and CH or CRF.

**Conclusions:**

The study suggests that age and CCT are strongly associated with CH and CRF, and that the latter is also influenced by AL and IOP. However, the variables studied could explain only 25% and 43% of the measured variation in CH and CRF, respectively, suggesting other factors also affect the values of these measurements.

## Background

Knowledge of the cornea’s biomechanical properties is important for a wide variety of applications within ophthalmology. Corneal biomechanical variations are known to affect the accuracy of intraocular pressure measurements [1,2], may be used to identify early corneal disease [3,4], and may assist with predicting refractive outcomes following corneal refractive surgery [5]. It has also been suggested that corneal biomechanical properties may reflect globe biomechanics and thus give an indication of the susceptibility of developing glaucomatous damage [6,7].

Until recently, most investigations evaluating corneal biomechanics were based on ex vivo tissue [8,9]. However, the Reichert Ocular Response Analyzer (ORA; Reichert Ophthalmic Instruments, Buffalo, NY, USA) has facilitated an in vivo measurement of aspects of corneal biomechanical properties. The device measures the central corneal response to indentation by a rapid jet of air and provides two metrics of corneal biomechanics, corneal hysteresis (CH) and the corneal response factor (CRF). It is thought that CH predominantly reflects the viscous dampening properties of the cornea whilst CRF, a metric empirically derived to be strongly correlated with central corneal thickness (CCT), is thought to be most associated with the cornea’s elastic response [10]. However, how these metrics relate to conventional biomechanical measurements is still relatively unclear. Studies examining the ORA biomechanical metrics have suggested associations with age, CCT and IOP [11,12]; however, some suggest no association with these parameters.

The purpose of this study was to evaluate the relationships between ORA generated corneal biomechanical characteristics and ocular dimensions, age and IOP in a cohort of normal eyes. Understanding the relationships between ORA measures and ocular dimensions may help better elucidate the significance of, and applications for, the metrics it produces.

## Methods

This was a prospective cross-sectional observational study. The study had the approval of the local research ethical committee (REC; London-City Road and Hampstead REC and Riverside REC, London, United Kingdom) and informed consent, according to the tenets of the Declaration of Helsinki, was obtained from each participant prior to examination. Study participants were recruited from staff, spouses and friends of patients attending Moorfields Eye Hospital, London, UK, or St. Luc University Hospital, Université Catholique de Louvain, Belgium. Data was collected between October 2008 and April 2011. Participants underwent a complete ophthalmic investigation, including visual field testing with the Humphrey Visual Field Analyser (HFA: Zeiss Humphrey Systems, Dublin, California, USA) SITA standard threshold 24–2 strategy. Participants were excluded from the study if they had any signs of corneal pathology, corneal astigmatism ≥2 dioptres or a history of incisional or intraocular surgery, a visual field defect, suspicious optic disc appearance, IOP ≥ 30 mmHg, a history of diabetes or a family history of glaucoma in a first degree relative. Soft-contact lens wearers were required to remove their lenses at least 24 hours prior to study participation; rigid contact lens wearers were excluded from the study. Of eligible participants, only one randomly chosen eye was measured for the study.

Study participants first had axial length (AL) and corneal curvature (CC) measurements made with the IOLMaster (version 3.01, Carl Zeiss Meditech AG, Jena, Germany). Prior to instillation of topical anaesthesia, participants underwent ORA measurements and three good quality waveform scans, defined as having symmetry in height between the two peaks of the waveform, were recorded and the mean value used in subsequent analysis.

Following instillation of topical corneal anaesthesia (proxymetacaine hydrochloride 0.5% with fluorescein sodium 0.25%), IOP measurements were made using both the Goldmann applanation tonometer (GAT) and the dynamic contour tonometer (DCT). Two GAT IOP and three DCT IOP measurements were made in a randomised order. Only DCT measurements with a 'quality’ reading of 1, 2 or 3 were accepted and the first DCT reading was discarded in accordance with manufacturer’s guidance. A minimum two-minute interval was left between IOP measurements with each device to minimise the tonographic effects of repeated tonometry measurements [13]. The mean of two IOP readings, taken with each of the two instruments, was calculated for each participant and was used in the analyses. Measurements of CCT, using an ultrasound pachymeter (Altair, Optikron 2000, Roma, Italy), were made at the end of the visit; the average of 3 readings was recorded. All measurements were performed by one of four investigators (AK, AS, DS or SP).

Based on previous pilot data from an unrelated dataset, it was calculated that a sample of 194 eyes was required to achieve a correlation between CH and age of rho = -0.2, with 80% power at the p < 0.05 level.

### Data analysis

Multivariate statistical analyses are effective tools to identify and explore the relationships between a response variable and several predictor variables. In this study, the response variable of interest was either CH or CRF and the predictor variables of interest were age, CC, CCT, IOP and AL. The theoretical assumptions of standard multiple linear regression (MLR) analysis limit its application to explore the relationships between CH, CRF and the predictors for this study as the predictor variables are correlated, a statistical phenomenon known as multicollinearity. In circumstances where there is multicollinearity in the predictors, partial least squares linear regression (PLSLR) offers a robust alternative to MLR [14]. Like MLR, the objective of PLSLR is to describe the relationships between the response and predictor variables.

For this study, two PLSLR models were fitted to a subset of the data under investigation. The 'calibration’ dataset consisted of a proportion (80%) of participants randomly selected from the complete sample used to construct a predictive model for CH and CRF. The number of components in each PLSLR model was chosen using the 'leave-one-out method’ [15]. The predictive performance of each model was then tested on the data excluded from the calibration data, which consisted of the remainder of participants. As the predictor variables have differing units of measurement, PLSLR models were also fitted using scaled predictor variables (where each predictor was weighted by dividing it by its standard deviation) in order to evaluate the relative impact of each variable on the response.

## Results

Data were collected from 212 participants and the demographics of the cohort are presented in Table 1. The majority of participants were of Caucasian ethnicity (n = 167), followed by Indian (n = 29), Far-East Asian (n = 9) and African/Afro-Caribbean (n = 7).

**Table 1:** Demographics of study cohort

**Total cohort n = 212**	**Mean**	**SD**	**Range**
Eye (left/number)	96		
Sex (male/number)	95		
Age (years)	50.4	19.0	19.0 to 92.6
AL (mm)	23.8	1.1	21.4 to 28.7
CC (mm)	7.7	0.3	7.1 to 8.6
Corneal astigmatism (dioptres)	0.8	0.5	0.0 to 4.0
CCT (microns)	550	31	490 to 633
GAT (mmHg)	14.8	3.3	6.0 to 25.5
DCT (mmHg)	16.2	2.6	9.7 to 25.0
ORA IOPcc (mmHg)	15.5	3.6	8.7 to 29.0

(Key: AL = axial length, CC = corneal curvature, CCT = central corneal thickness, GAT = Goldmann applanation tonometry, DCT = dynamic contour tonometry, IOPcc = ORA corneal compensated IOP).

### Correlations between CH, CRF and other parameters

As some of the data were non-normally distributed, Spearman’s rank correlation test was used to evaluate correlations between parameters (Table 2). ORA corneal biomechanical parameters were positively correlated with ORA IOPcc and GAT IOP, and to a lesser extent with DCT IOP. CH and CRF were negatively associated with age, axial length and corneal curvature, and positively correlated with CCT.

**Table 2:** Correlation table showing Spearman’s rho and significance values for parameters

**Spearman’s rho coefficient (p)**	**Age (yrs)**	**AL (mm)**	**CC (mm)**	**CCT (microns)**	**GAT IOP (mmHg)**	**DCT IOP (mmHg)**	**IOPcc (mmHg)**	**CH (mmHg)**
AL (mm)	**-0.19**^**†**^							
	*(0.007)*							
CC (mm)	-0.01	**0.45**^**†**^						
	*(0.87)*	*(<0.001)*						
CCT (microns)	0.12	-0.04	-0.01					
	*(0.09)*	*(0.54)*	*(0.98)*					
GAT IOP (mmHg)	-0.06	-0.01	-0.08	**0.20**^**†**^				
	*(0.41)*	*(0.84)*	*(0.27)*	*(<0.01)*				
DCT IOP (mmHg)	0.06	0.07	-0.01	**0.20**^**†**^	**0.78**^**†**^			
	*(0.39)*	*(0.34)*	*(0.93)*	*(<0.01)*	*(<0.001)*			
IOPcc (mmHg)	**0.15**^**†**^	0.07	0.10	**0.15**^**†**^	**0.56**^**†**^	**0.57**^**†**^		
	*(0.03)*	*(0.28)*	*(0.16)*	*(0.02)*	*(<0.001)*	*(<0.001)*		
CH (mmHg)	**-0.19**^**†**^	**-0.16**^**†**^	**-0.16**^**†**^	**0.36**^**†**^	**0.24**^**†**^	**0.20**^**†**^	**-0.26**^**†**^	
	*(<0.01)*	*(0.02)*	*(0.02)*	*(<0.001)*	*(<0.001)*	*(<0.01)*	*(<0.001)*	
CRF (mmHg)	**-0.22**^**†**^	**-0.23**^**†**^	**-0.16**^**†**^	**0.47**^**†**^	**0.28**^**†**^	**0.24**^**†**^	**-0.11**^**†**^	**0.74**^**†**^
	*(0.001)*	*(0.002)*	*(<0.01)*	*(<0.001)*	*(<0.001)*	*(<0.001)*	*(0.01)*	*(<0.001)*

*(Key: AL = axial length, CC = corneal curvature, CCT = central corneal thickness, GAT IOP = Goldmann applanation tonometry intraocular pressure, DCT IOP = dynamic contour tonometry intraocular pressure, IOPcc = Ocular response analyzer corneal compensated intraocular pressure, CH = corneal hysteresis, CRF = corneal response factor,*^*†*^*= significant at the p < 0.05 level).*

Previous work has shown the DCT to be less affected by variations in corneal biomechanical properties [16]; consequently, for this study, the DCT IOP measurement was used as a surrogate measure of 'true’ IOP. Thus, in total, the impact of five predictor variables on CH and CRF were considered.

### PLSLR analysis: corneal hysteresis and corneal response factor models

**Figure 1:**
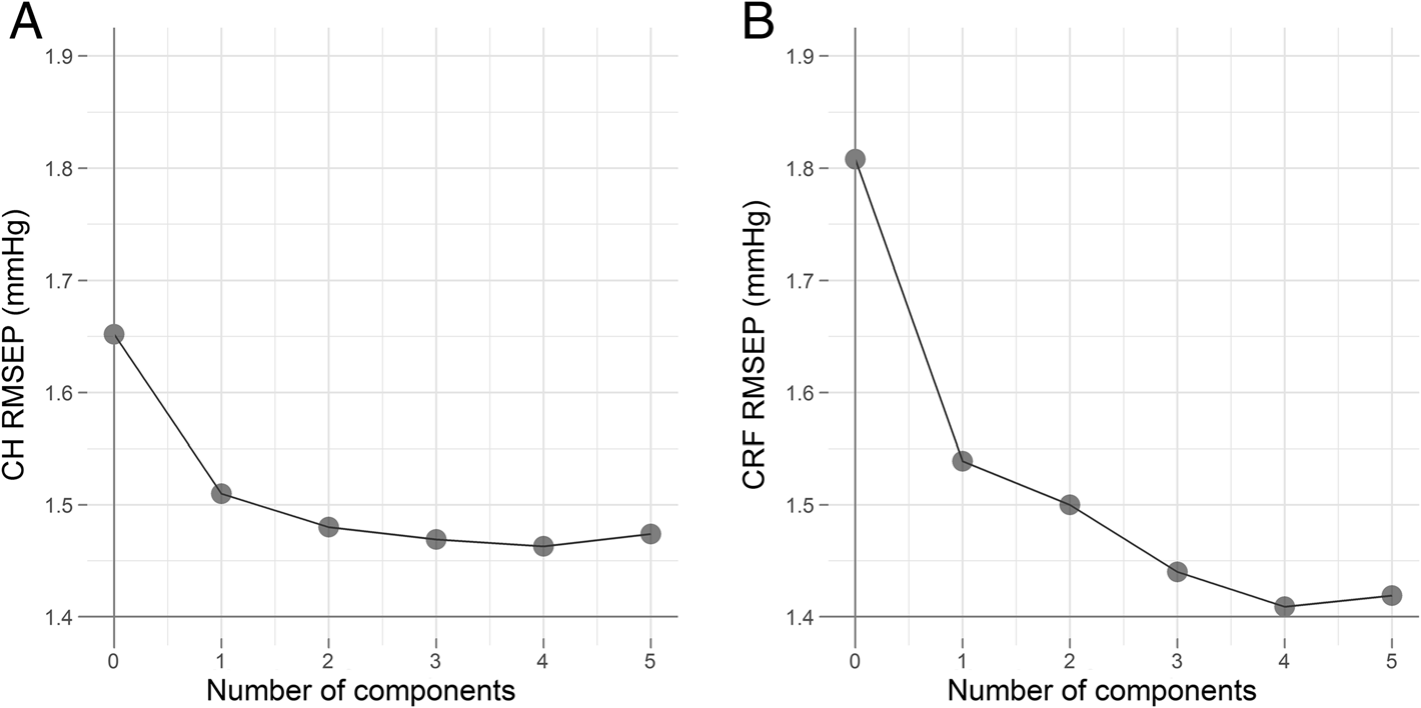
**Cross-validated RMSEP curves for PLSLR models.** These graphs illustrate the effect of the number of components on the precision of the CH **(A)** and CRF **(B)** regression models. In both models, the prediction error was minimized using four components.

All statistical analyses were carried out in R, a freely available statistical package ( http://www.r-project.org/). A random selection of 169 participants was used to calibrate the PLSLR model, and the results of cross-validation (using the leave-one-out method) are illustrated in Figure 1; the root mean squared error of prediction (RMSEP) is plotted against the number of components included in the fitted model. Four components minimise the RMSEP in the CH model (RMSEP = 1.46; Figure 1A), and four components minimise the RMSEP in the CRF model (RMSEP = 1.42; Figure 1B). Therefore, four components were employed in the CH and CRF PLSLR predictive models, respectively.

For the CH model, four components explained 25% of the variance in CH. For the CRF model, four components explained 43% of the variance in CRF.

The coefficients for the CH and CRF PLSLR models are shown in Tables 3 and 4, respectively. The data illustrates that age and CCT exerts a strong influence on CH and CRF, with the later also being associated with axial length and IOP.

**Table 3:** Coefficients of PLSLR predictive model for CH in the calibration dataset

**Predictor variable**	**PLSLR coefficient (non-scaled)**	**PLSLR coefficient (scaled)**	**P value**
AL (mm)	-0.23	-0.22	0.05
CC (mm)	-0.02	-0.02	0.85
CCT (microns)	0.02	0.62	<0.0001
Age (years)	-0.03	-0.55	<0.0001
DCT IOP (mmHg)	0.09	0.24	0.05

The scaled coefficients illustrate that CCT and age have the most significant impact on CH. (Key: AL = axial length, CC = corneal curvature, CCT = central corneal thickness, DCT IOP = dynamic contour tonometry intraocular pressure).

**Table 4:** Coefficients of PLSLR predictive model for CRF in the calibration dataset

**Predictor variable**	**PLSLR coefficient (non-scaled)**	**Scaled PLSLR coefficient**	**P value**
AL (mm)	-0.39	-0.37	<0.01
CC (mm)	-0.03	-0.04	0.74
CCT (microns)	0.03	0.89	<0.0001
Age (years)	-0.03	-0.60	<0.0001
DCT IOP (mmHg)	0.18	0.46	<0.01

The scaled coefficients illustrate that CCT has the greatest impact on CRF, followed by age, IOP and AL. (Key: AL = axial length, CC = corneal curvature, CCT = central corneal thickness, DCT IOP = dynamic contour tonometry intraocular pressure).

**Figure 2:**
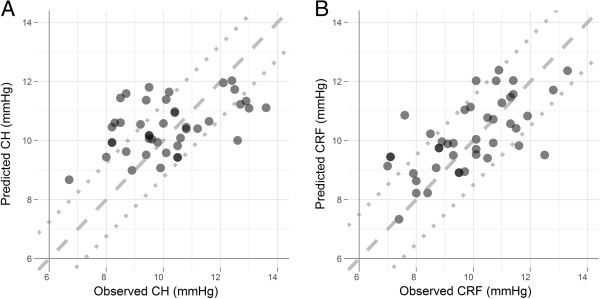
**Scatterplot showing the prediction performance of the PLSLR in the test dataset.** Graphs show the PLSLR prediction performance of CH **(A)** and CRF **(B)**. The dashed line represents the line of unity. If the prediction model was 100% accurate, all data points would fall on this line. The dotted lines indicate the 95% confidence limits of the Normal distribution; these are ±1.24 and ±1.50 for CH and CRF, respectively. Compared with the CH model, predicted values of CRF are close to observed values, indicating that the studied variables can explain a large proportion of the variation in CRF measurements.

Finally, the accuracy of the models to predict CH and CRF was tested using the independent validation data made up of 43 normal participants. The predicted value for each response is plotted against the observed value in Figures 2. The CH prediction model produced an r^2^ value of 0.16 and a RMSEP equal to 1.44 (see Figure 2A), while the CRF prediction model gave rise to an r^2^ value of 0.38 and a RMSEP equal to 1.24 (see Figure 2B).

## Discussion

The results of this study suggest that ORA-generated metrics of corneal biomechanical properties in non-glaucomatous eyes are correlated with age, CCT and, in the case of CRF, AL. The relationships between these variables are complex and the predictor variables do not explain all the variation in CH or CRF measurements.

The data were analysed with PLSLR analysis since the predictor variables were correlated. In PLSLR, components (known as 'latent variables’) are extracted from the predictor variables by maximising the covariance between the predictor variables and the response variable.

It has been suggested that CH represents the viscoelastic capacity of the cornea, that is, the cornea’s ability to dampen and dissipate applied energy. Hysteresis is dependent on the relative contributions of both elasticity and viscosity, and it has been shown that alterations in either component will have very different and sometimes opposing effects on measured hysteresis [17]. Our finding that CH reduces with age corroborates the findings of experimental ex vivo studies that show an increase in collagen cross-linking with age [18], which result in a reduction in the viscosity of the cornea and thus an increase in the 'stiffness’ of the structure [19,20]. The data are also in agreement with previous clinical work evaluating the effect of age on ORA measured corneal biomechanical properties [11,12,21]. Taken together, these findings suggest that aging results in an overall reduction in the dampening capacity of the cornea.

Our study also agrees with previous findings that CH is positively associated with CCT [11,21]. This is unsurprising as one might expect a thicker cornea to have a greater viscous dampening capacity. Recent work has also found that CH reduces with increasing axial length [22], and it has been suggested that these findings are indicative of altered biomechanics in axially myopic eyes. However, other reports have found no association between axial length and CH [23]. In the present study, Spearman’s correlation test suggested that CH was negatively associated with both axial length and corneal curvature; however, in the PLSLR model, these parameters were not significant (although axial length approached significance). These findings may be a reflection of the ethnic differences in the cohorts studied; our participants were predominantly of Caucasian ethnicity, whilst those in the other studies were of Far East Asian origin. Surprisingly, Spearman’s correlation test also suggested that CH was positively associated with DCT IOP and GAT IOP; however, in the PLSLR model the association between DCT IOP and CH only approached significance. This suggests that the univariate association was either a spurious finding, or as a result of correlations with other predictor variables that were subsequently dealt with in the PLSLR model. Further work is required to establish the significance of relationships that may exist between axial length, IOP and CH.

CRF was also found to increase with CCT and reduce with age. CRF was intended to quantify the overall corneal viscoelastic resistance to indentation and was developed to be strongly associated with CCT [24]. The fact that CRF reduces with age is counterintuitive, as it might be expected that the increase in corneal 'stiffness’ resultant from an age-related increase in corneal collagen cross-linking would result in an increased resistance to deformation; however, our results do agree with previous findings [25]. The CRF represents a metric of corneal resistance to a near instantaneous indentation force applied axially. Our data suggest that the assumption that CRF reflects overall corneal rigidity may be an oversimplification and that other factors need to be considered when interpreting its value.

In the PLSLR model, CRF was also found to be positively associated with IOP. The cornea is hyperelastic; thus, the 'stretching’ of the cornea under high IOP conditions will render it a 'stiffer’ structure. Of interest is the finding that a lower CRF was associated with longer axial length, which was highlighted in the PLSLR model. This agrees with some [26] but not others’ [22] findings. It is not clear what this relationship between CRF and axial length biomechanically represents. One may postulate that if CRF represents overall corneal rigidity, since longer eyes may have altered scleral biomechanics [27] a reduction in CRF may be an expected finding, in that a reduced corneal rigidity would be associated with a reduced scleral rigidity. However, further work is required to establish the significance of the relationship and how corneal biomechanics relate, if at all, to scleral biomechanics.

Of note is the proportion of variance in corneal biomechanical measurements predicted by the studied parameters when all the inter-relationships are considered. In the calibration PLSLR model, only 25% of the variation in measured CH could be explained by the variables studied, which reduced even further when applied to the test dataset. Whilst part of this may indicate true inter-individual variations in CH measurements, there may be other explanations for this observation. It is possible that this reflects a reduced signal-to-noise ratio in the CH measurement which masks the true effect of the predictors studied [28]. Furthermore, there may be significant and as yet unmeasured variables that effect the CH measurement. These may include the degree of corneal indentation and the rate and maximal level of external air pressure application, all of which have been shown experimentally to affect hysteresis [17,29]. In contrast, approximately 43% of CRF variation could be explained by the variables studied. This suggests that the CRF measurement can be characterised by CCT, age, IOP and AL. However, a large proportion of the measurement variation cannot be explained by these variables and may represent a combination of actual inter-individual CRF differences, measurement noise and other as yet undetermined factors.

## Conclusion

In conclusion, this study of corneal biomechanics in normal eyes describes the complex interactions between ocular characteristics and ORA metrics, and finds that both age and CCT are significantly associated with CH and CRF. However, only a proportion of the variation in both these ORA metrics could be described by the ocular characteristics measured, implying that there are other elements contributing to the CH and CRF measurement.

Finally, condensing corneal biomechanical measures to a single summary metric will never completely describe the cornea’s properties. Recently, investigators evaluating the ORA applanation signal have found that variations in specific signal elements are better descriptors of the corneal response to indentation particularly following refractive surgery procedures [30,31]. Further work is required to establish the validity of these new parameters and how they relate to more conventional biomechanical measures.

## Competing interests

The authors declare that they have no competing interests.

## Authors’ contributions

AK: study design, data acquisition, statistical analysis, data interpretation, manuscript writing and final approval. RAR: statistical analysis, data interpretation, manuscript critique and final approval. AS: data acquisition, manuscript final approval. SP: data acquisition, manuscript critique and final approval. DS: data acquisition, manuscript final approval. DGH: study design, data interpretation, manuscript critique and final approval. All authors read and approved the final manuscript.

## Pre-publication history

The pre-publication history for this paper can be accessed here:

http://www.biomedcentral.com/1471-2415/14/11/prepub
